# Nitrogen-fixing root nodules elicited by rhizobial potassium ion transporter *Smkup1*: senescence and autophagy

**DOI:** 10.3389/fpls.2026.1749975

**Published:** 2026-02-26

**Authors:** Maria G. Semenova, Teodoro Coba de la Peña, Aleksandra N. Petina, Tatiana Ivashina, Elena E. Fedorova

**Affiliations:** 1 Timiryazev Institute of Plant Physiology, Russian Academy of Science, Moscow, Russia; 2 Centro de Estudios Avanzados en Zonas Áridas (CEAZA), La Serena, Chile; 3 Skryabin Institute of Biochemistry and Physiology of Microorganisms, Federal Research Center Pushchino Scientific Center for Biological Research, Russian Academy of Sciences, Pushchino, Russia

**Keywords:** autophagy, nitrogen fixation, rhizobium potassium transport, root nodule, senescence, stress

## Abstract

With the aim to elucidate the interdependence between potassium transport by the host plant in nodule cells and potassium transport in bacteroids, a null mutant of rhizobial potassium ion transporter *Smkup1* was created and investigated. The mutation, according to cytological analysis, has not caused specific aberrations in the root nodules’ anatomy and ultrastructure, but a significant induction of the expression of host plant and rhizobial genes involved in the stress response was observed. At the same time, an opposite trend was observed for genes of the autophagy pathway that have shown a significant downregulation of expression. To identify the mechanisms of interplay between autophagy and senescence in the root nodule, an *in silico* analysis of protein–protein interactions of positive (Beclin 1) and negative (NAC1, BAK1) regulators of autophagy was performed. The resulting networks allowed the predictions of interacting proteins putatively linking symbiotic interactions, autophagy, stress, programmed cell death (PCD), and senescence. Based on these data, we hypothesized that modulation of the expression of these genes in the root nodule could be the way to extend the root nodule’s lifespan and the duration of the nitrogen fixation process.

## Introduction

Symbiotic nitrogen fixation in the root nodules of legumes is one of the important sources of fixed nitrogen for the biosphere. Nitrogen-fixing bacteria temporary reside in the infected cells of root nodule symplast (Oldroyd et al., 2011) forming a colony, putatively with some extracellular property (Luo et al., 2022). Simultaneously with the growth of the root nodule, intracellular bacteria gain access to host cell resources, colonize the host cells, and begin to fix atmospheric nitrogen through the induction of the enzyme nitrogenase (Beringer, 1974; Parniske, 2018; Roy et al., 2020; Coba de la Peña et al., 2018).

The termination of symbiosis, known as “root nodule senescence”, is manifested as a lysis of the infected cells (Berrabah et al., 2023). At the cellular level, the life span of infected cells is quite short, around 3–6 weeks, and the root nodule is highly sensitive to suboptimal environmental conditions (Chakraborty and Harris, 2022). The reasons for the rapid termination of symbiosis have not yet been elucidated in detail, but the short lifespan of infected cells compared to the lifespan of root cells from which the nodules are formed may indicate the suboptimal conditions of the infected cells and the induction of the termination processes specific for the root nodule (Wojciechowska et al., 2018; Sauviac et al., 2022; Berrabah et al., 2023). A high vulnerability of root nodule tissue may be caused by some pre-existing defects in the ion balance of the root nodule (Fedorova et al., 2021; Trifonova et al., 2022). Therefore, the study of factors involved in root nodule homeostasis requires further research. Potassium is one of the major ions involved in the adaptive responses of plants to abiotic stresses (Rubio et al., 2020; Zhang et al., 2022; Wakeel and Ishfaq, 2021). It is essential for controlling the osmotic status as well as the cation–anion balance of the cell. The availability of K^+^ to the cell depends on the transport and concentration of K^+^ on both sides of the membrane. Potassium transporters in plants are involved in K^+^ uptake or release as well as storage in vacuoles and translocation of ions between tissues and organs (Ragel et al., 2019; Fedorova et al., 2021, Luo et al., 2022).

With the aim to elucidate the interdependence between potassium transport mediated by host plant ion transporters in nodule cells (Domínguez-Ferreras et al., 2009; Zhang et al., 2022) and potassium transport in bacteroids, we have created and analyzed a bacterial null mutant of bacterial potassium ion transporter *Smkup1*. To elucidate the possible reaction of symbiotic cells, the expression of genes induced during root nodule senescence and autophagy induction was analyzed since the termination of symbiosis involves the lysis of the nodule cells and the bacteria within them (Berrabah et al., 2023; Wleklik and Borek, 2023). To identify the putative mechanisms of symbiosis termination, we conducted an *in silico* analysis of protein/protein interactions of several regulators of autophagy and senescence.

## Materials and methods

*Medicago truncatula* Gaertn. cv. Jemalong A17 plants were grown according to Limpens et al. (2004). *Sinorhizobium meliloti* strain Sm2011 was used for nodulation. *M. truncatula* seeds were sterilized and germinated according to Limpens et al. (2004). Seedlings were sown in sand/vermiculite (1:1) mixture supplemented with Fahraeus medium and inoculated with *S. meliloti* 2011–3 days after sowing. *M. truncatula* plants were grown in a growth chamber (20°C, 16 h day light, 60%–70% relative humidity), supplemented by halogen lamps (PPF 1580 мкмоль/м²/с). The light intensity on the level of plants is 140–160 μ mo. Nodules were harvested after 8 weeks.

To analyze the growth of control (Sm2011) and mutant (*Smkup1*) strains of rhizobia, the density of the bacteria culture was measured over a period of days of growth in LB liquid culture; the suspension density was determined using a GENESYS™ 40/50 vis/UV– vis spectrophotometer. The samples have been measured in triplicates from the set of nine analytical 15- mL tubes and grown on a shaker for 3 days. On each day, the three tubes have been measured.

The nodule weight per plant was measured after 8 weeks of cultivation. The sample size in both experiments was 20 plants per analysis.

The analysis of gene expression was performed by using real-time quantitative PCR (qPCR) with a 7300 Real Time PCR System (PE Applied Biosystems) with SYBR 455 Green Supermix (Evrogen, Russia). The genes *Mtc27*, *MtGAPDH*, and *SMc00128* were used as endogenous controls for the plant host and for bacteria, respectively (Coba de la Peña et al., 2008). The ΔΔC_T_ method (Pfaffl, 2001) was applied for PCR relative quantification. For PCR analysis, four to six biological replications were used.

Total RNA was isolated from 8-week-old nodules of control plants (inoculated with wt Sm2011) and of plants inoculated with the *Smkup1* mutant using the RNeasy Plant Minikit (Qiagen, USA) and retrotranscribed into cDNA using SKO22L kit (Evrogen, Russia). A Thermo Scientific NanoDrop microvolume spectrophotometer was used for RNA concentration measurements. The RNA purity and integrity quality have been estimated by using the A260/A280 ratio.

Gene-specific primers were designed using the PRIMER 3-PLUS software (Untergasser et al., 2007). Light and electron microscopy, respectively, have been used to analyze the anatomy and ultrastructure of the nodules. The nodules were fixed and embedded in LR White as described (Fedorova et al., 2021), observed by using a light microscope, and analyzed using a LIBRA120 electron microscope.

### Construction of *S. meliloti* Kup1 transporter deletion mutant

The *S. meliloti* strain Sm2011, carrying a deletion in the *kup1* gene (SM2011_c00873), was obtained by gene replacement technique (Sambrook and Russell, 2001). For this purpose, the recombinant plasmid pJQ/Δ*kup1*::Km was constructed as follows: Two fragments containing the 5′- and 3′- ends of *kup1* with upstream and downstream genomic sequences were amplified from the genomic DNA of Sm2011 and sequentially cloned into the suicide vector pJQ200SK (Gm^R^*sacB*) (Quandt and Hynes, 1993) to give the pJQ/Δ*kup1* plasmid. The primers used are listed in **Supplementary Table S1**. The deletion was marked by the insertion of a 1.26-kb Km^R^-cassette from pUC4K (Pharmacia, Sweden) into the BamHI-site of pJQ-Δ*kup1* to yield pJQ/Δ*kup1::*Km. The resulting plasmid contains a 1, 122-bp deletion (corresponding to the removal of 374 aa from 633 aa of the protein) in the coding sequence of the *kup1* gene with flanking genomic sequences. The target plasmid was introduced into *S. meliloti* Sm2011Rif^R^ cells by conjugation from *E. coli* strain S17-1 (Simon et al., 1983) with primary selection of merodiploid clones (Suc^S^Km^R^Gm^R^ phenotype). Double crossovers were selected on LB plates containing sucrose (5%) and Km (20 μg/mL). Individual Km^R^ clones were tested for sensitivity to gentamicin (10 μg/mL). The allelic exchange was confirmed by using PCR.

### *In silico* protein–protein interactions analysis

The predicted protein–protein interaction (PPI) networks were obtained using the STRING (Search Tool for the Retrieval of Interacting Genes/Proteins) database (http://www.string-db.org). The obtained networks represent both functional and/or physical protein–protein interactions predicted in *M. truncatula*, and they are based on both known and/or predicted interactions. The associations among proteins are derived from various channels: text mining, experiments, databases, coexpression, neighborhood, gene fusion, and co-occurrence. The PPI networks for proteins of interest were obtained with medium to high (0.4 or higher) confidence scores.

## Results

**Figure 1:**
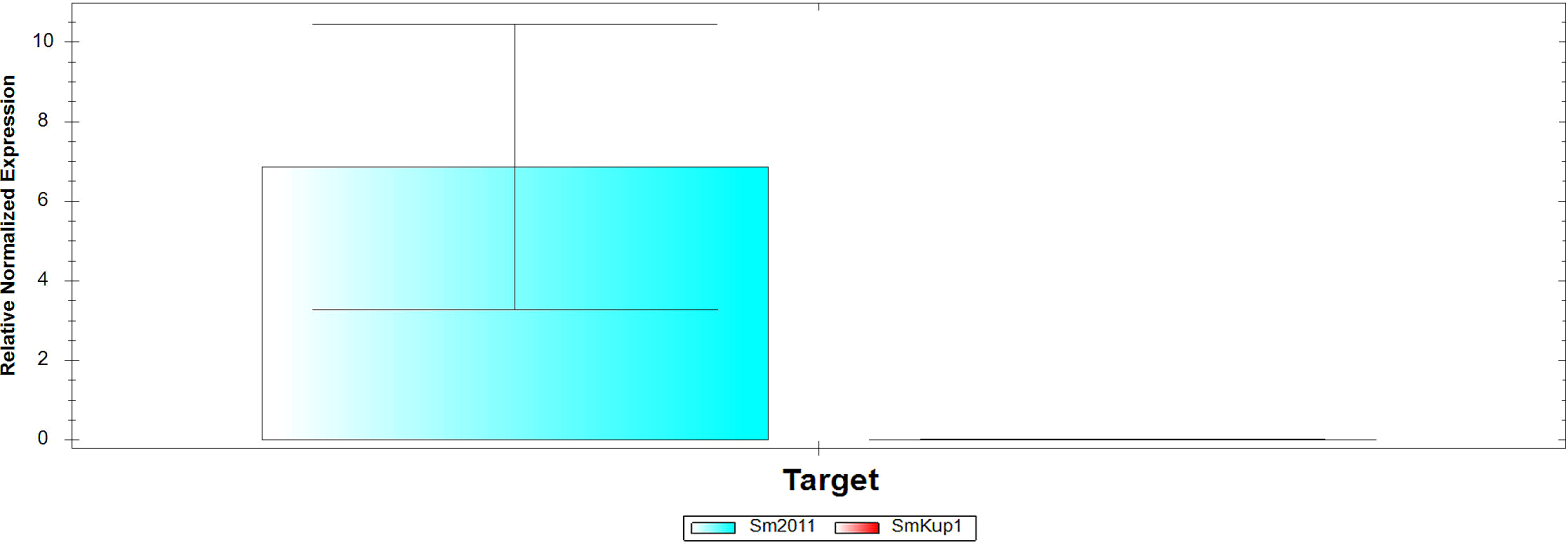
Gene expression in control Sm2011 and in *Smkup1* mutant strains. Analysis was performed by using real-time qPCR.

The mutation *Smkup1* was verified using the quantitative PCR method (**Figure 1**), which demonstrated the absence of *kup1* gene expression on the mutant background.

To elucidate the biological characteristics of the mutant *Smkup1* in symbiosis, an analysis of its growth dynamics in bacterial culture, an analysis of nitrogen fixation ability of root nodules, and an analysis of the expression of macro- and microsymbiont genes that respond to stress and the genes of autophagy pathway were conducted. The effect of mutation on the growth of rhizobia was analyzed in a bacterial culture grown for 3 days on LB medium (**Table 1**).

**Table 1:** Dynamics of wild -type (Sm2011) and mutant bacteria (*Smkup1*) growth in liquid culture.

Bacteria/culture growth time (h)	Optical density of bacterial culture			
	0 h	24 h	48 h	72 h
Sm2011 (WT)	0.4 ± 0.0	0.607 ± 0.08	0.755 ± 0.01 *	0.969 ± 0.09 *
*Smkup1*	0.4 ± 0.0	0.588 ± 0.05	0.615 ± 0.06	0.760 ± 0.08

Asterisks (*) indicate significant statistical differences between the two strains of rhizobia.

The dynamics of wild-type (Sm2011) and mutant bacteria (*Smkup1*) growth in liquid culture. Asterisks (*) indicate significant statistical differences between the two strains of rhizobia. The analysis of bacterial culture growth has shown a significant difference between control strain Sm2011 and *Smkup1* mutant after 48 and 72 h of growth (*) (**Table 1**). The mutant strain growing in culture significantly lags behind the control strain by growth rate.

The impact of *Smkup1* mutation on the growth of root nodules was assessed based on the mass of root nodules per plant after cultivation for 8 weeks post-sowing (**Table 2**).

**Table 2:** Weight of root nodules elicited by *Sm2011* and S*mkup1* mutant (mg).

Sm2011	S*mkup1*
0.205 ± 0.032	0.204 ± 0.022

Based on the analysis of nodule growth, no significant differences in root nodule mass between the wild type and the mutant was observed; that means that the root nodule growth *per se* was not negatively affected by *Kup1* loss of function.

Phenotypically, the root nodules also did not show signs of rapid senescence such as the formation of green-colored zones as a result of the breakdown of leghemoglobin.

### Analysis of root nodule ultrastructure

**Figure 2:**
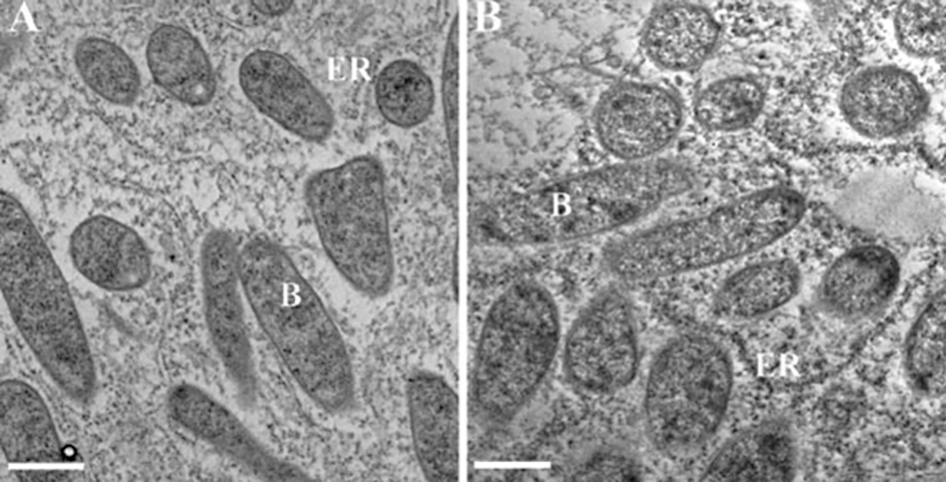
Ultrastructure of infected cells in the fixation zone of the nodules, elicited by *Smkup1* mutant **(A)** and wild-type nodules **(B)**. B, bacteroid; ER, endoplasmic reticulum. Bar, 1 μm.

According to the cytological analysis, the nodules elicited by *Smkup1* bacteria have not displayed any pronounced defects of nodule histology or anatomy. Neither did it cause the formation of a specific early senescent phenotype (**Supplementary Figure S1**). Electron microscopy of root nodules formed upon inoculation with the control rhizobium strain and the mutant strain did not reveal a specific phenotype; the light and electron microscopy analysis of nodule structure, respectively, are presented in **Figure 2** and **Supplementary Figure S1**. The ultrastructure has not demonstrated the sites of symbiosis termination or rapid lytic elimination of the microsymbiont (**Figure 2**). The light microscopy images of nodules presented in **Supplementary Figure S1** also do not show any drastic alteration in root nodule anatomy or rapid senescence.

The absence of pronounced anatomical changes in the root nodules apparently reflects the fact that a mutation in a single rhizobial gene, as it appears in this case, has not immediately led to the induction of a lytic clearance of bacteroids in the infected cells and host cell lysis that often is a typical outcome in suboptimal conditions of host plant, root nodules, or incompatible symbiotic partners (Sauviac et al., 2022).

**Figure 3:**
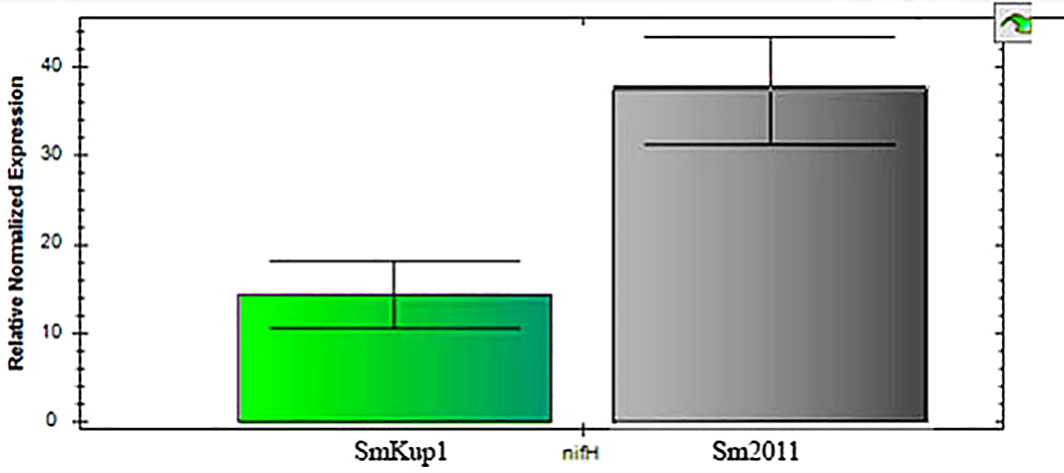
Expression of *NifH* gene in root nodules elicited by *Smkup1* and the control strain Sm2011. Gene expression was performed by using real-time qPCR.

The expression of the *NifH* gene, the indicator of nitrogen fixation ability, in root nodules elicited by mutant *Smkup1*and the control strain Sm2011 did not show a significant difference in the level of gene expression (**Figure 3**).

**Figure 4:**
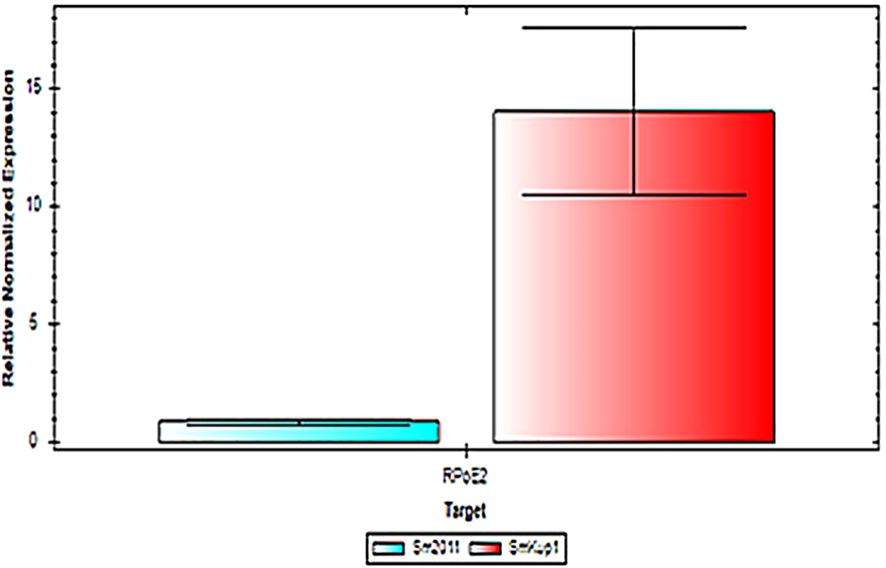
Comparative expression of bacterial stress marker *RPoE2* in root nodules elicited by control strain Sm2011 and mutant strain *Smkup1*, analyzed by using qPCR.

In order to evaluate the stress level in mutant-induced nodules, the comparative expression of genes associated with micro- and macrosymbiont stress and senescence was analyzed. The gene selected for the diagnostics for *S. meliloti* was *RPoE2* (Sauviac et al., 2007; da-Silva et al., 2017; Belfquih et al., 2022), an envelope stress response sigma factor that is involved in the general stress response in *S. meliloti*. The gene *RPoE2* is upregulated under oxidative, saline, and osmotic stress and by microaerobic conditions (da-Silva et al., 2017). For the diagnostics of stress response of the host plant (*M. truncatula*), the gene *MtCP5*, a cysteine protease-encoding gene used as a marker of nodule senescence, was selected (Sauviac et al., 2022). It was shown that the expression of the *RPoE2* gene is significantly upregulated in nodules elicited by *Smkup1* mutant in comparison to control nodules elicited by the control strain *Sm2011* (**Figure 4**), which confirms the stress conditions of microsymbiont in root nodules elicited by the mutant strain.

**Figure 5:**
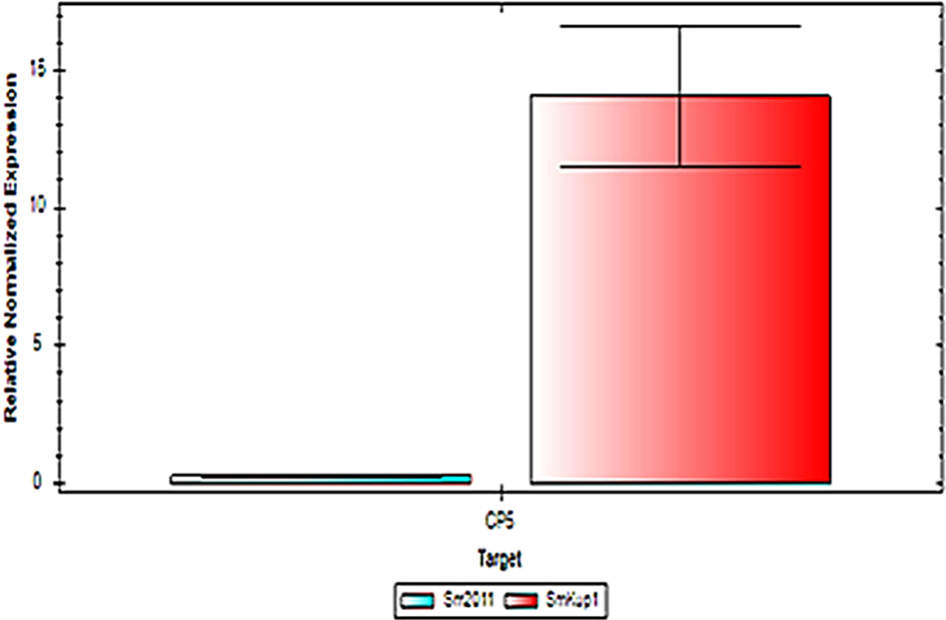
Expression of host plant stress response gene *MtCP5* in root nodules elicited by control strain *Sm2011* and mutant strain *Smkup1*. Analysis was performed by using real-time qPCR.

The expression analysis has shown a significant upregulation of gene *MtCP5* in mutant-induced nodules (**Figure 5**), which reflects the host cells’ stress response.

It was assumed that, despite the absence of visible cytological aberrations, the nodules have shown the impact of mutation manifested in the upregulation of expression of stress- and senescence-related genes of both the bacterial partner and the host plant (**Figures 4**, **5**).

Biologically, it can be anticipated that the presence of thousands of bacteria inside of the eukaryotic cell may lead to an accelerated senescence and the lytic clearance of the infected cells. According to our previous study, in normal conditions, autophagy genes are expressed at relatively high levels and the expression is maintained through all developmental zones of wild-type root nodules (Semenova et al., 2024). However, the presence of “classical “ autophagosomes has not been shown in the root nodules of the wild type.

**Figure 6:**
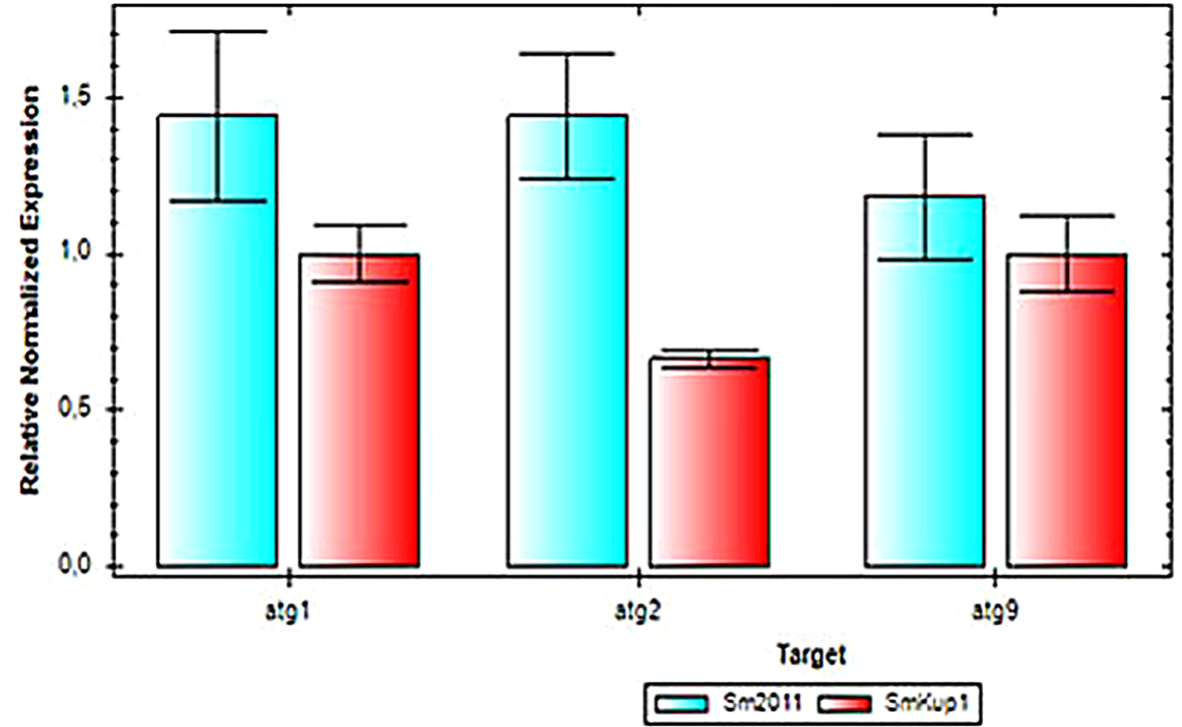
Expression of autophagy genes in root nodules elicited by control strain Sm2011 and mutant strain *Smkup1*. Analysis was performed by using real-time qPCR.

**Figure 7:**
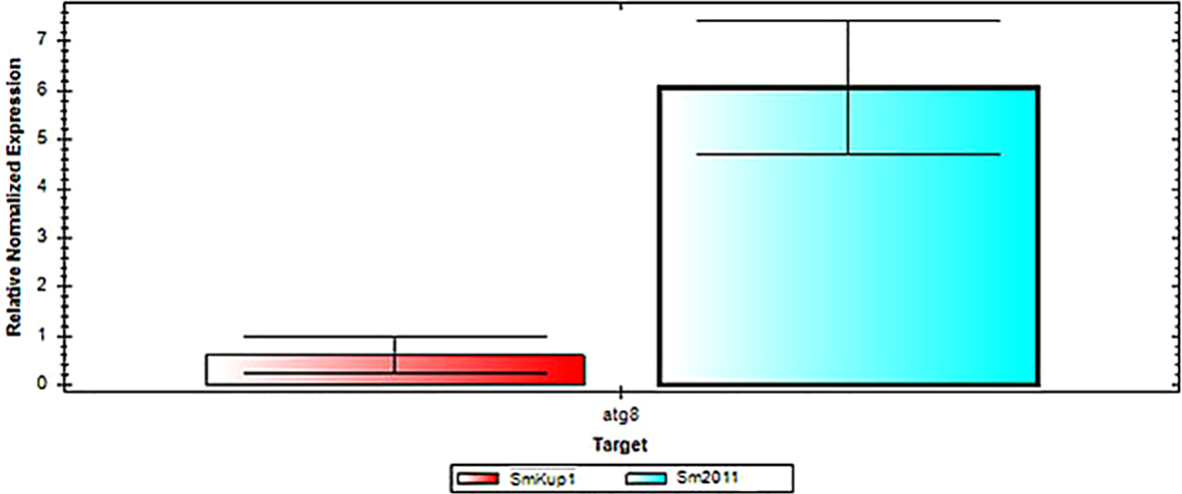
Comparative expression level of *ATG8* gene in root nodules elicited by the mutant strain *Smkup1* and by control strain Sm2011. The difference in expression level between both types of nodules is significant. Analysis was performed by using real-time qPCR.

With the aim to diagnose the putative ways of autophagy regulation in the nodules, we have performed an analysis of the expression of autophagy pathway genes (Agbemafle et al., 2023; Semenova et al., 2024) in root nodules elicited by the mutant *Smkup1* strain. For the analysis, genes of autophagy pathways *ATG1*, *ATG2*, *ATG8*, and *ATG9* were analyzed (**Figures 6**, **7**). A tendency to decrease in the expression of low-level expressed autophagy genes *ATG1*, *ATG2*, and *ATG9* was observed in root nodules elicited by the mutant (**Figure 6**). A statistically significant downregulation of the high-level expressed gene *ATG8* was detected in mutant-induced nodules in comparison to nodules induced by the wild-type strain (**Figure 7**). This pattern of expression was in counterphase with the expression of genes associated with the stress response of host plant and microsymbiont (**Figures 4**, **5**).

To elucidate the relationships between the pathways of autophagy regulation during the root nodule development, genes involved in the regulation of autophagy in root nodules have been selected (Cadwell et al., 2025; Estrada-Navarrete et al., 2016, Dong et al., 2021; James et al., 2025). The expression levels of these genes in the developmental zones of *M. truncatula* root nodules were estimated *in situ* using the portal Symbimix (Roux et al., 2014) (**Table 3**). According to the level of expression, it was observed that positive regulators of autophagy *Beclin1*, *SnF*, *WRKY20*, and *Dhh1* (genes that are upregulated by starvation) were highly expressed in mature infected cells, which may indicate an energy limitation in this developmental zone. The genes involved in autophagosome development and maturation (*TGA9*, *WRKY24*, and *WRKY33*) were expressed in the apical part of the nodule. Genes involved in autophagy inhibition (*SOC1*, *PP2A*, *NAC1*, *TORC*, and *BAK1*) were upregulated in different nodule zones, which putatively hints for development-stage-specific regulation (**Table 3**).

**Table 3:** Level of expression of positive and negative regulators of autophagy in the developmental zones of *M. truncatula* root nodule.

Gene	Gene ID or locus name	Functions	Expression level				
		Positive regulators of autophagy	FI	FIId	FIIp	IZ	ZIII
*Beclin1*	NC_053044.1 Mt0017_00537	Positive; central regulator of energy and nutrient perception	14	11	23	18	**33**
*SnF*	NC_053047.1 Mt0025_10284	Positive; induces transcription of ATG under starvation and ABA signaling	13	13	14	23	**38**
*TGA9*	Mt0003_00116	Positive; induces atg8 lipidation, autophagosome maturation	**47**	16	9	15	13
*WRKY20*	Mt0010_00868	Positive; autophagosome maturation, cargo recognition	26	18	9	20	**28**
*WRKY24*	Mt0008_10932	Positive; autophagosome maturation, cargo recognition	**29**	12	10	25	24
*WRKY33*	Mt0045_10227	Positive; autophagy receptor, regulates atg8 lipidation, PI3P effector	**46**	9	3	31	11
*Dhh1*	NC_053047.1 Mt0025_10284	Positive; activated under starvation, interacts with ATG1/ULK1 kinase complex and ATG8, facilitates translation of ATG1 and ATG13 mRNA	18	15	17	22	**24**
*SOC1*	Mt0001_01425	Positive; upregulation, renders the autophagy process more active	**55**	12	7	12	14
		Negative regulators of autophagy					
*PP2A*	Mt0009_00048	Negative; serine/threonine protein phosphatase dephosphorylates ATG13 upon starvation	23	21	**25**	16	15
*NAC1*	Mt0008_10932	Negative; *Medicago truncatula* NAC domain-containing protein A signal for nitrogen deficiency, abiotic stress, control PCD	**45**	38	0	11	6
		Suppresses autophagy to avoid excessive degradation					
*TORC*	NC_053048.1 Mt0026_10261	Negative; inhibits autophagy under high sugar levels; in case of downregulation, exhibits constant active autophagy	15	14	18	19	**33**
*BAK1*	Mt0023_10383	Negative; phosphorylates ATG18a, which suppresses autophagy	**30**	28	11	15	15

Expression levels data (relative read distribution among zones, %) were obtained from the Symbimics database. The highest level of expression in nodule developmental zones is highlighted in bold.

FI, meristem; FIId, distal infection zone; FIIp, proximal infection zone; IZ, interzone II–III; ZIII, nitrogen-fixing zone, mature zone.

**Figure 8:**
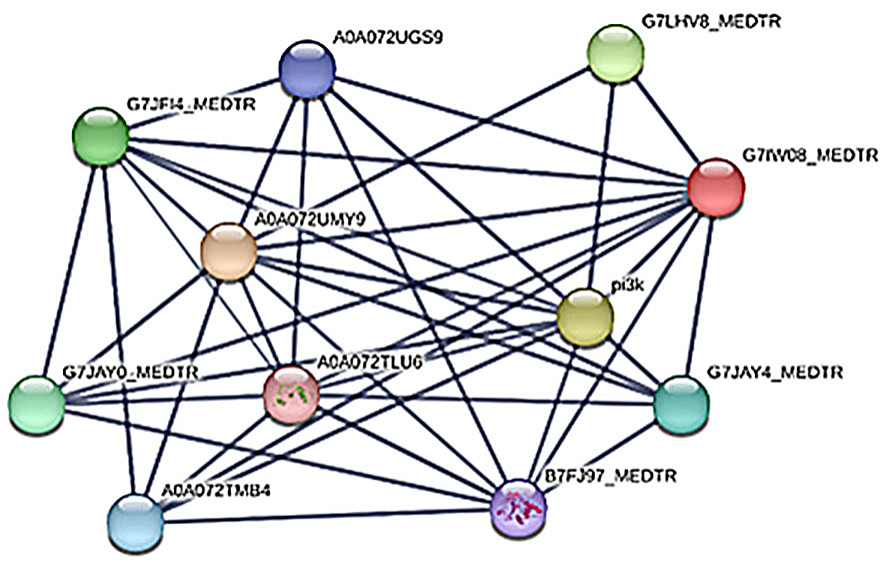
Protein/protein interaction network for the positive regulator of autophagy Beclin-1. The thickness of the lines indicates the strength of data support. Beclin-1 protein is named in the network with its UniProt identifier G7IW08_MEDTR, and its corresponding node is colored in red. Predicted interacting proteins are named with their UniProt identifiers and are explained in the text.

**Figure 9:**
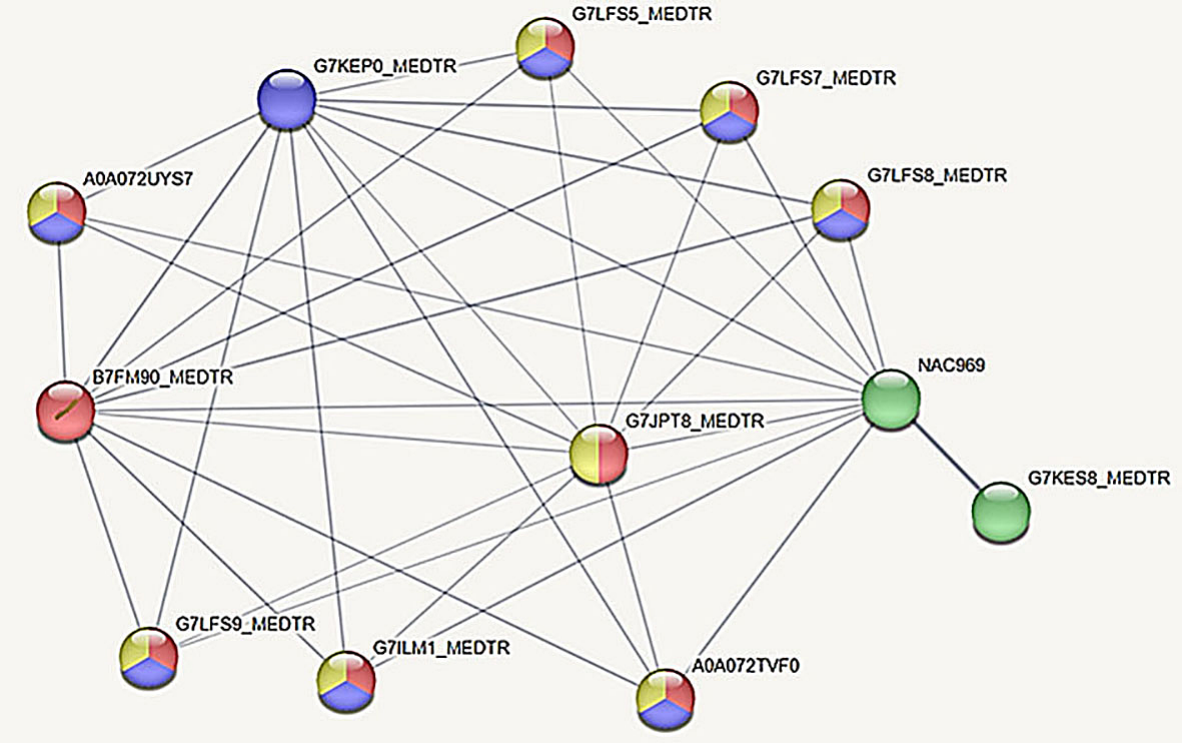
Protein/protein interaction network for *M. truncatula* NAC domain-containing protein (Yu et al., 2023). Interacting proteins involved in symbiotic interaction, programmed cell death (PCD), stress response, and senescence were selected. Nodes corresponding to proteins are named with their corresponding Uniprot identifiers. NAC969 is the UniProt identifier of the NAC1 protein. The thickness of the lines indicates the strength of data support. Color code and network proteins are described in the text. Network was obtained with the STRING software.

**Figure 10:**
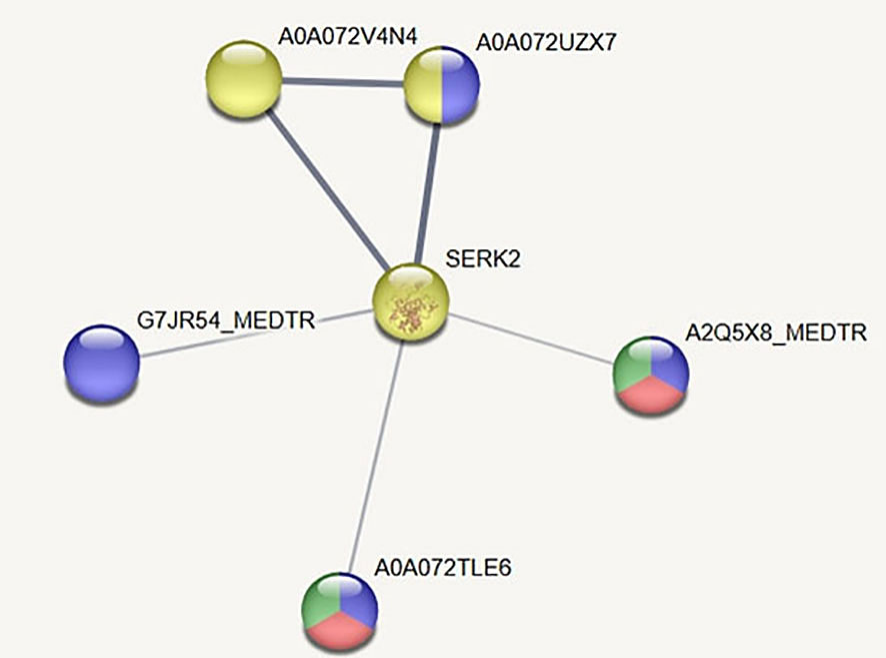
Protein/protein interaction network for a negative regulator of autophagy BAK1. Interacting proteins involved in the negative regulation of defense response, negative regulation of cell death, plant–pathogen interaction, and defense response to bacterium were selected. Nodes corresponding to proteins are named with their corresponding Uniprot identifiers. SERK2 is the UniProt identifier of BAK1. The thickness of the lines indicates the strength of data support. Color code and network proteins are described in the text. Network was obtained with the STRING software.

With the aim to detect putative regulation pathways for genes of autophagy in root nodule, some genes from the list of positive and negative regulators of autophagy (**Table 3**) were selected for an *in silico* analysis of protein/protein interactions (**Figures 8**–**10**). The analysis was performed for the central positive regulator of autophagy Beclin1, a negative regulator of autophagy NAC1, and a negative regulator of autophagy BAK1. The predicted protein–protein interaction (PPI) networks were obtained using the STRING database (http://www.string-db.org).

The proteins from the network related to Beclin-1 (Mt0017_00537, UniProt identifier G7IW08_MEDTR) include the following: a nodule isoform of a Pi3k phosphatidylinositol 3-kinase, which is an autophagy-related protein (G7JF14_MEDTR); a tyrosine kinase protein (G7JAYO_MEDTR); a calcium-dependent kinase protein (G7JAY4_MEDTR); two serine/threonine kinase proteins (AOAO72TMB4 and AOAO72UGS9); the autophagy-related protein 3 (B7FJ97_MEDTR); the ubiquitin-like protein ATG12, involved in cytoplasm to vacuole transport (AOAO72TLU6); and a UV- radiation-resistance-associated -like protein (G7LHV8_MEDTR) (**Figure 8**).

NAC domain-containing protein (NAC1, Mt0008_10932), a negative regulator of autophagy, is a transcription factor involved in several pathways, including abiotic stress response and symbiotic nodule senescence (de Zélicourt et al., 2012; Dong et al., 2021).

When analyzing putative protein–protein interactions of NAC1 (UniProt identifier NAC969 and with the node in green color in **Figure 9** using STRING software with medium confidence score of 0.4 or higher), it was found that most interacting proteins are related to response to auxin and other hormones, nitrogen deficiency, and responses to organic substances and chemicals. It is interesting to note that from this protein network interaction, several genes are related to biological processes involved in symbiotic interaction (nodes with blue color), cellular response to stress (nodes with yellow color), programmed cell death (nodes with red color), and nodule senescence (nodes in green color) (**Figure 9**).

G7KEP0_MEDTR is a NAC domain-containing protein that is a putative transcription factor involved in symbiotic interaction. G7KES8_MEDTR is a STAY-GREEN protein (MtSGR) involved in nodule development and senescence (Zhou et al., 2021). Network proteins involved in PCD include G7JPT8_MEDTR (an Executer 1 chloroplastic protein), B7FM90_MEDTR (a putative cytochrome b6f complex subunit), and seven proteins with membrane attack complex component/perforin (MACPF) domains (A0A072TVF0, A0A072UYS7, G7LFS5_MEDTR, G7LFS7_MEDTR, G7LFS8_MEDTR, G7LFS9_MEDTR, and G7ILM1_MEDTR).

BAK1(Mt0023_10383) is a BRASSINOSTEROID INSENSITIVE 1-associated receptor kinase 1, and it is a negative regulator of autophagy (Zhang et al., 2022). When analyzing putative protein–protein interactions of BAK1 (Uniprot identifier SERK2) using STRING software with medium confidence score (0.4 or higher), it was found that most network interacting proteins are involved in several pathways, including the regulation of hormone (brassinosteroids, auxin)-mediated signaling pathways and levels, signal transduction, and response to oxygen-containing compounds. It is interesting to note that, from this protein network interaction, several genes are involved in the negative regulation of cell death (nodes with red color), defense response to bacterium (nodules with blue color), negative regulation of defense response (nodes with green color), and plant–pathogen interaction (nodes with yellow color) (**Figure 10**).

G7JR54_MEDTR is a putative Sec7 domain, mon2, dimerization, and cyclophilin-binding domain-containing protein involved in defense response to bacterium. A2Q5X8_MEDTR and A0A072TLE6 are LRR receptor-like kinases; they are involved in defense response to bacterium, and they also are negative regulators of defense response and negative regulators of cell death. A0A072UZX7 is a LRR receptor-like kinase protein involved in defense response to bacterium and in plant–pathogen interactions. A0A072V4N4 is also a LRR receptor-like kinase involved in plant–pathogen interactions. SERK2 is a Ser/Thr protein kinase also involved in plant–pathogen interactions.

In order to examine the relationships between protein–protein interactions and the expression of the genes involved, an analysis of the expression of gene Beclin1, the positive regulator of autophagy, was conducted on wild-type strain 2011 (A) and the mutant strain *Smkup1* (**Supplementary Figure S2**). The expression of gene Beclin1 in root nodules elicited by wild-type strain 2011 was significantly higher, that of a mutant strain *Smkup1*.

## Discussion

In the process of legume–rhizobia symbiosis, a colony of living bacteria is maintained for a long time (up to 4 to 5 weeks) in the symplast of living root nodule cells, which is an exceptionally rare phenomenon for any eukaryote. The maintenance of the colony leads to changes in the biological characteristics of the infected cell and may be causal for the infected cell’s short lifespan (Chakraborty and Harris, 2022)— for example, the potassium content diminishes in infected cells of *Medicago truncatula* nodules due to the mislocation of host plant channels MtAKT1 and MtSKOR/GORK involved in intracellular potassium transport (Fedorova et al., 2021).

The role of each partner in potassium transport in root nodules is not fully understood (Domínguez-Ferreras et al., 2009; Fedorova et al., 2021; Li et al., 2024), so we have analyzed the involvement of the bacterial partner of the symbiosis using the mutant of a bacterial potassium transporter *Smkup1.* The mutation of one from several bacterial transporters has not caused a structural damage of the root nodule or rapid senescence of the symbiosomes. However, despite the absence of visible aberrations, root nodules have a high level of expression of genes involved in stress response (**Figures 4**, **5**). At the same time, the expression of genes from ATG group responsible for the induction of autophagy was downregulated in comparison with wild-type-induced root nodules (**Figures 6**, **7**).

Autophagy is a housekeeping process that is induced in the cells of eukaryotic organisms under unfavorable conditions, such as lack of energy, limited access to food, or the presence of microorganisms in the cells. It is assumed that the introduction of bacteria into any eukaryotic cell triggers a protective response that manifests as the induction of autophagy processes. Autophagosomes, the lytic organelles induced in the cell for digestions of parts of the cytoplasm or cell organelles, are formed under stress conditions or defense against bacteria that have entered the cell (Cadwell et al., 2025). In light of this, the role of autophagy is recognized as essential housekeeping processes (Agbemafle et al., 2023; Estrada-Navarrete et al., 2016; Liu et al., 2020; Zheng et al., 2025).

The studies investigating autophagy processes induced by microorganisms mainly are focused in Gram-negative mammalian pathogens, such as *Salmonella* and *Coxiella* (Cadwell et al., 2025). Animal pathogens are able to form so-called bacteria-containing vacuoles, which consist of a host cell membrane containing one or more bacteria, that allows pathogens to survive within a cell for several hours and avoid immediate elimination (Cadwell et al., 2025). The membrane of bacteria-containing vacuoles is marked by small GTPases of the RAB family (RAB7 and RAB24) and specific polyphosphoinositides, that determine membrane/organelle identity and the membrane fusion (Liu et al., 2020; Cadwell et al., 2025). The existence of pathogen-containing vacuoles is terminated by the fusion with autophagosomes, vacuole-like structures with lytic properties. The process of fusion is coordinated by RAB GTPase RAB7 and effectors of homotypic fusion and protein sorting (HOPS) complex VPS11 and VPS15.

Structurally, bacteria-containing vacuoles have much in common with symbiosomes in root nodule cells, symbiosome membranes are also recruiting small GTPase RAB7, and SNARE proteins (Gavrin et al., 2014). However, HOPS effectors VPS11 and VPS15 recruitment to symbiosome membranes is inhibited due to the strong downregulation of the expression of these effectors in infected root nodule cells (Gavrin et al., 2014), making symbiosomes temporarily immune to the autolysis.

In this regard the conditions that prevent or temporarily inhibit the autophagic clearance of bacteria in root nodule infected cell may be of a quite important for the symbiotic relationships.

According to *in situ* expression, the genes involved in the autophagy pathway are expressed in all zones of the root nodule (Semenova et al., 2024); however, autophagosomes in their “classical form” (similar to structures found in animal cells infected with bacteria) were not diagnosed in root nodule cells apart of the mutant DNF1 nodules, that demonstrates that the presence of autophagosomes in infected cells is an extremely rare phenomenon (Wang et al., 2010). The autophagy clearance in root nodule infected cells elicited by mutant strain *Smkup1* is rather inhibited than upregulated, that hints at the opportunities for maintaining the existence of a bacterial colony even in unfavorable conditions. Although infected cell experiences suboptimal sugar levels, insufficient levels of essential ions like potassium, a low oxygen level, it is able to support a colony of nitrogen-fixing bacteria up to 6–8 weeks (Belfquih et al., 2022; Ayala-García et al., 2025; Zhou et al., 2021) albeit at the cost of its own lifespan.

The high expression of such genes as *Beclin1*, *SnF* and *Dhh1*, according to *in situ* analysis (**Table 3**) key regulators of the autophagy process hints the starvation environment in the nitrogen fixation zone of the nodule as well as an induction of autophagy. Quite interesting is the expression in the root nodule cells of *WRKY20*, the gene involved in autophagosome maturation, taking into account that in these cells the autophagosomes have never been found. The autophagosomes’ membrane formation depends on the host membranes, mainly those of the endoplasmic reticulum. It should be remembered that the membranes of symbiosomes are also formed from the host cell endoplasmic reticulum (Bellucci et al., 2017; Behnia and Munro, 2005, Fedorova, 2023). How to reconcile and explain the relatively high level of autophagy gene expression in the root nodule and the absence of “classical “ autophagosomes in the cells remains unclear. The mechanisms involved in preventing the autophagic removal of symbiosomes and death of infected cells, which are induced in the root nodule, are also not yet understood.

We can hypothesize that the regulation of autophagy in root nodules, at least in part, may depend on the conformation of proteins rather than genes. To identify potential pathways for autophagy regulation in root nodules, we decided to conduct an *in silico* analysis of protein–protein interactions based on the genes of autophagy pathways that we have already identified.

Quite interesting is the role of a positive regulator of autophagy Beclin1 that, according to the analysis of protein/protein interactions, is associated with the ubiquitin-like protein ATG12 (UniProt identifier AOAO72TLU6), which belongs to an autophagy pathway and is involved in cytoplasm- to-vacuole transport (**Figure 8**), the processes that are partly inhibited in infected cells (Gavrin et al., 2014).

The analysis of the interaction network for *M. truncatula* NAC1, a putative transcription factor of symbiotic interaction, has shown interacting proteins involved in programmed cell death (PCD) and stress response as well as a STAY-GREEN protein (UniProt identifier G7KES8_MEDTR) that regulates nodule development and senescence (Zhou et al., 2021) (**Figure 9**).

The analysis of BRASSINOSTEROID INSENSITIVE 1-associated receptor kinase 1 (BAK1) has shown interacting proteins involved in the negative regulation of defense response to bacteria and negative regulation of cell death (**Figure 10**). The role of BAK1 in the prevention of bacteria elimination in symbiotic cells may be of major importance and needs further research.

Data concerning protein–protein interactions in infected cells of root nodules and certain similarities between the biogenesis of the symbiosome and the formation of the autophagosome permit us to hypothesize that the autophagy pathway may be co-opted into the processes of symbiosis formation and that the regulation of this process may be quite important in stress conditions.

This could indicate putative neofunctionalization of autophagy genes in a symbiotic process that may be involved at a later stage of intracellular accommodation, after the formation of nitrogen-fixing symbiosomes, and be a useful tool for symbiosome maintenance regulation.

## Data Availability

The original contributions presented in the study are included in the article/**Supplementary Material**. Further inquiries can be directed to the corresponding author.
