# Supplementary material for: Nitrogen-fixing root nodules elicited by rhizobial potassium ion transporter Smkup1: senescence and autophagy
Source: Front Plant Sci. 2026 Feb 26;17:1749975. doi: 10.3389/fpls.2026.1749975 (PMC12979505; doi:10.3389/fpls.2026.1749975)

**Semenova et al., Suplementary**

**Primers used for PCR analysis**

| Gene name | Primers |
| --- | --- |
| *SmNifH* | (L) 5’-TCCACGACCTCCCAAAATAC-3’ |
|  | (R) 5’- CGCACTTGATGCCTCTGTAA-3’ |
| *RpoE2* | (L) 5’-GGACACGATCATGAAGGCCT -3’ |
|  | (R) 5’-ACAAATCGAGGTGCTACCGC -3’ |
| *MtATG1* | (L) 5’-TGGTTTTGCACGGTCTCTCA -3’ |
|  | (R) 5’-TCTGGGTGTCAGTTGTGCTC -3’ |
| *MtATG9* | (L) 5’-CTCAACAAGGGTGTGCTTGC -3’ |
|  | (R) 5’-AATTGCAGCAAAGCCACCTG -3’ |
| *MtATG2* | (L) 5’-CAGGATGTGGTGAAGCTGGT-3’ |
|  | (R) 5’-ACTCGCCCCAAAATCAGGTT-3’ |
| *MtATG8* | (L) 5’-TATGTCGCTCCCTCATTGGC-3’ |
|  | (R) 5’-ACACCGTCTCTCTACACTCTCT-3’ |
| *MtCP5* | (L) 5’-TGGCCTCTAGATTCCCCCAA-3’ |
|  | (R) 5’-CCCAAATTGCAGGGCTTTCC-3’ |
| Beclin-1 | (L) 5’- AGGCCAAAGATGTCCTCAGC-3’ |
|  | (R) 5’- ACTGTTGCTGGTGTCTGTGA-3’ |
| Reference gene | |
| *MTC 27* | (L) 5’-CACCCAAACTAGATGCAGAGAAACA-3’ |
|  | (R) 5’- CAAAGAATTGAAGGTCCTTGAGC-3’ |
| *SMc00128* | (L) 5’-ACGAGANCGAGATCGCCATT-3’ |
|  | (R) 5’- CGAACGAGGTCTTCAGCATGA-3’ |

The primers used for the construction of the mutant

| SMkup1_SacI 5'- | AAGAGAGAGCTCGTCTATCGAAGCGACAC-3' |
| --- | --- |
| SMkup1_BamHI-1 5'- | AAGGATCCGGGGGGTAACCAACTTCAA-3' |
| SMkup1_BamHI-2 5'- | AAGGATCCGGGGACGGACGGCAAACACACACG-3' |
| SMkup1_BamHI-1 5'- | AAGGATCCGGGGGGTAACCAACTTCAA-3' |
| SMkup1_mf 5'- | TCTTCATCGGCGGCGACGCGAT-3' |
| Smkup1_mr 5'- | GCCCGACTTCCTCCTGAAGAGGC-3' |

# The deleted region in the Kup1 protein is marked in red.

# MSQLSAPATPGVENARRLLVLALGSVGVVYGDIGTSPLYAFREALRPVSHDGVTDVEIIGLISLMIWALTIIVTIKYVLFLLRADNQGEGGTLSLLALLMKTANGHTAILFFMGIAGAALFIGDAMITPALSVLSAVEGLKLVTPALSDYVVPIAVVILLLLFTVQSKGTAAVSNFFGPITLIWFVVMGTIGFVHIADDLSIFRAFNPYYAASFLFNEGYVGIVVLGAVFLTVTGAEALYADLGHFGRRPIQWAWFTVVFPALTLNYLGQGAFVLKNPEAMSDPFFLMFPKWALLPAVILATAATIIASQAVITGAFSLTRQAIHLGFLPRMAIFHTSETHTGQIYLPNVNTLLMFGVMALVFLFGSSEALATAYGISVTGAMVVTTVLSFEFLRMRWNWPTWWAAGALLPLFVLEFVFLGANMLKIHDGGYVPILIAATFIVIMWTWKRGTAILHAKTRHIDIPLASFIKSVERQSEHAPVSVTGTAIFLTSDPESTPAALLHNIKHNHVLHQQNFILTIRTANTPKVPKEERVSVRRLSERFTLLEMKFGFMETQNVSQALGLFRKSGLKFDIMSTSFYLGRRKLVPDAQSGMPHWQDRLFIALANAAIDPSDYFRLPTNRVVELGSHVII

NAC domain-containing protein 1, sequence and the protein

ATGGAGAGTAGTGCAAGCTCTGAACTCCCTCCTGGCTTTAGATTTCATCCAACTGATGAGGAACTAATTGTGCATTACCTTTGTAATCAAGCTACATCAAAGCCATGCCCTGCATCTATCATACCAGAAGTTGATATCTATAAATTTGATCCATGGGAATTGCCTGATAAATCTGAGTTTGAGGAAAATGAATGGTATTTCTTTAGCCCAAGAGAAAGAAAGTATCCAAATGGGGTGAGGCCTAATAGAGCAACTTTGTCTGGATATTGGAAAGCTACTGGCACAGACAAGGCAATCAAAAGTGGATCAAAGCAAATTGGTGTGAAGAAATCTTTGGTATTTTACAAAGGTAGACCACCAAAGGGTGTCAAAACTGATTGGATTATGCATGAGTACAGATTGATTGGATCACAAAAACAAACTAGCAAGCATATTGGATCCATGAGGCTAGATGACTGGGTTCTATGCAGGATCTATAAGAAGAAGCACATGGGAAAAACATTGCAGCAAAAAGAGGATTATTCAACACATCAATTTAATGATTCTATAATAACTAATAATGATGATGGTGAACTAGAAATGATGAACCTTACAAGGAGTTGTTCACTTACTTATCTTTTGGATATGAATTACTTTGGTCCAATCTTATCTGATGGTTCAACTTTGGATTTTCAAATCAACAATTCCAATATTGGAATTGACCCCTATGTAAAACCTCAGCCTGTTGAAATGACCAACCATTATGAAGCAGATTCACATAGTAGCATCACCAATCAGCCAATATTTGTGAAACAAATGCATAATTATTTAGCATAA

>NP_001411868.1 NAC domain-containing protein 1 [Medicago truncatula] (NAC969)

MESSASSELPPGFRFHPTDEELIVHYLCNQATSKPCPASIIPEVDIYKFDPWELPDKSEFEENEWYFFSPRERKYPNGVRPNRATLSGYWKATGTDKAIKSGSKQIGVKKSLVFYKGRPPKGVKTDWIMHEYRLIGSQKQTSKHIGSMRLDDWVLCRIYKKKHMGKTLQQKEDYSTHQFNDSIITNNDDGELEMMNLTRSCSLTYLLDMNYFGPILSDGSTLDFQINNSNIGIDPYVKPQPVEMTNHYEADSHSSITNQPIFVKQMHNYLA

When analyzing putative protein-protein interactions with medium confidence (0.4 or higher), and setting network interaction with 300 proteins, we found that most interacting proteins are related to response to auxin and other hormones, responses to organic substances and chemicals, and response to stimulus (see Gene Ontology figure).

>NM_001424939.1:7-822 Medicago truncatula NAC domain-containing protein 1 (LOC11445056), mRNA

ATGGAGAGTAGTGCAAGCTCTGAACTCCCTCCTGGCTTTAGATTTCATCCAACTGATGAGGAACTAATTGTGCATTACCTTTGTAATCAAGCTACATCAAAGCCATGCCCTGCATCTATCATACCAGAAGTTGATATCTATAAATTTGATCCATGGGAATTGCCTGATAAATCTGAGTTTGAGGAAAATGAATGGTATTTCTTTAGCCCAAGAGAAAGAAAGTATCCAAATGGGGTGAGGCCTAATAGAGCAACTTTGTCTGGATATTGGAAAGCTACTGGCACAGACAAGGCAATCAAAAGTGGATCAAAGCAAATTGGTGTGAAGAAATCTTTGGTATTTTACAAAGGTAGACCACCAAAGGGTGTCAAAACTGATTGGATTATGCATGAGTACAGATTGATTGGATCACAAAAACAAACTAGCAAGCATATTGGATCCATGAGGCTAGATGACTGGGTTCTATGCAGGATCTATAAGAAGAAGCACATGGGAAAAACATTGCAGCAAAAAGAGGATTATTCAACACATCAATTTAATGATTCTATAATAACTAATAATGATGATGGTGAACTAGAAATGATGAACCTTACAAGGAGTTGTTCACTTACTTATCTTTTGGATATGAATTACTTTGGTCCAATCTTATCTGATGGTTCAACTTTGGATTTTCAAATCAACAATTCCAATATTGGAATTGACCCCTATGTAAAACCTCAGCCTGTTGAAATGACCAACCATTATGAAGCAGATTCACATAGTAGCATCACCAATCAGCCAATATTTGTGAAACAAATGCATAATTATTTAGCATAA

>NP_001411868.1 NAC domain-containing protein 1 [Medicago truncatula] (NAC969)

MESSASSELPPGFRFHPTDEELIVHYLCNQATSKPCPASIIPEVDIYKFDPWELPDKSEFEENEWYFFSPRERKYPNGVRPNRATLSGYWKATGTDKAIKSGSKQIGVKKSLVFYKGRPPKGVKTDWIMHEYRLIGSQKQTSKHIGSMRLDDWVLCRIYKKKHMGKTLQQKEDYSTHQFNDSIITNNDDGELEMMNLTRSCSLTYLLDMNYFGPILSDGSTLDFQINNSNIGIDPYVKPQPVEMTNHYEADSHSSITNQPIFVKQMHNYLA

The sequence of the corresponding gene and corresponding protein are:

>NM_001424939.1:7-822 Medicago truncatula NAC domain-containing protein 1 (LOC11445056), mRNA (Zélicourt et al. Plant Journal 2012).

ATGGAGAGTAGTGCAAGCTCTGAACTCCCTCCTGGCTTTAGATTTCATCCAACTGATGAGGAACTAATTGTGCATTACCTTTGTAATCAAGCTACATCAAAGCCATGCCCTGCATCTATCATACCAGAAGTTGATATCTATAAATTTGATCCATGGGAATTGCCTGATAAATCTGAGTTTGAGGAAAATGAATGGTATTTCTTTAGCCCAAGAGAAAGAAAGTATCCAAATGGGGTGAGGCCTAATAGAGCAACTTTGTCTGGATATTGGAAAGCTACTGGCACAGACAAGGCAATCAAAAGTGGATCAAAGCAAATTGGTGTGAAGAAATCTTTGGTATTTTACAAAGGTAGACCACCAAAGGGTGTCAAAACTGATTGGATTATGCATGAGTACAGATTGATTGGATCACAAAAACAAACTAGCAAGCATATTGGATCCATGAGGCTAGATGACTGGGTTCTATGCAGGATCTATAAGAAGAAGCACATGGGAAAAACATTGCAGCAAAAAGAGGATTATTCAACACATCAATTTAATGATTCTATAATAACTAATAATGATGATGGTGAACTAGAAATGATGAACCTTACAAGGAGTTGTTCACTTACTTATCTTTTGGATATGAATTACTTTGGTCCAATCTTATCTGATGGTTCAACTTTGGATTTTCAAATCAACAATTCCAATATTGGAATTGACCCCTATGTAAAACCTCAGCCTGTTGAAATGACCAACCATTATGAAGCAGATTCACATAGTAGCATCACCAATCAGCCAATATTTGTGAAACAAATGCATAATTATTTAGCATAA

>NP_001411868.1 NAC domain-containing protein 1 [Medicago truncatula] (NAC969)

MESSASSELPPGFRFHPTDEELIVHYLCNQATSKPCPASIIPEVDIYKFDPWELPDKSEFEENEWYFFSPRERKYPNGVRPNRATLSGYWKATGTDKAIKSGSKQIGVKKSLVFYKGRPPKGVKTDWIMHEYRLIGSQKQTSKHIGSMRLDDWVLCRIYKKKHMGKTLQQKEDYSTHQFNDSIITNNDDGELEMMNLTRSCSLTYLLDMNYFGPILSDGSTLDFQINNSNIGIDPYVKPQPVEMTNHYEADSHSSITNQPIFVKQMHNYLA

**Supplementary Primers list:**

kup_SacI 5'-AAGAGAGAGCTCGTCTATCGAAGCGACAC-3'

kup_BamHI-1 5'-AAGGATCCGGGGGGTAACCAACTTCAA-3'

kup_BamHI-2 5'-AAGGATCCGGGGACGGACGGCAAACACACACG-3'

kup_PstI 5'-AACTGCAGCTCGTGCGCTGAAGGAT-3'

kup_mf 5'-TCTTCATCGGCGGCGACGCGAT-3'

kup_mr 5'-GCCCGACTTCCTCCTGAAGAGGC-3'

S
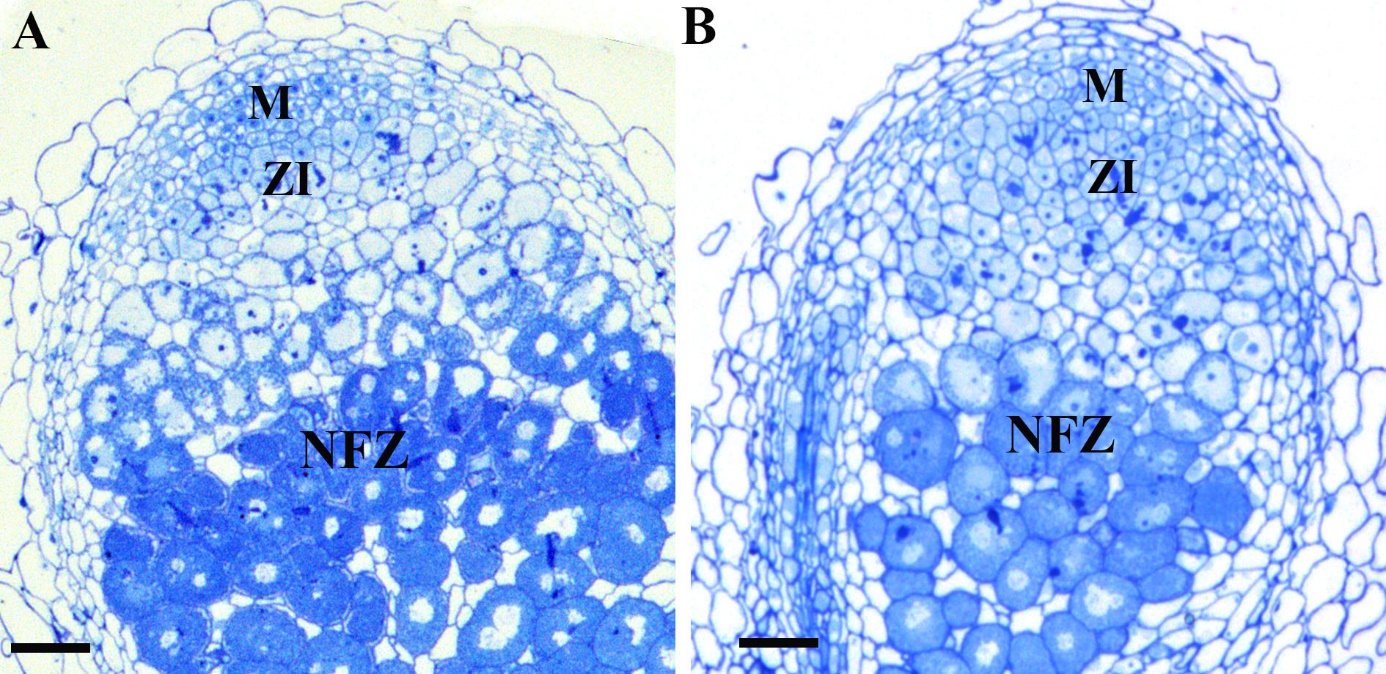


**B**

**A**

Supplementary Fig.1.Root nodules elicited by wt strain 2011 (A) and the mutant strain *Smkup1* (B).ZI- zone of infection, NFZ- nitrogen fixation zone. The bar: 100mm

Supplementary Fig. 2. The expression analysis of gene Beclin1, the positive regulator of autophagy on wt strain 2011 (A) and the mutant strain *Smkup1*(B)


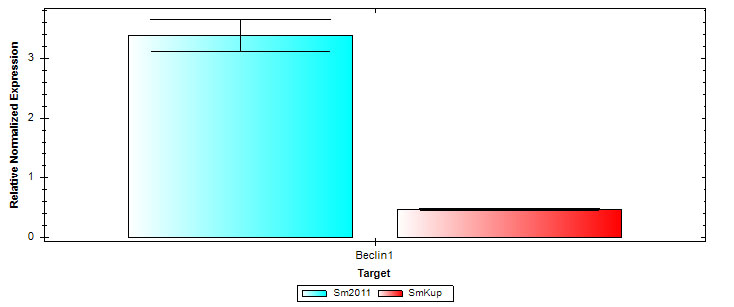

Supplement: Supplementary file 1 [file DataSheet1.docx]
